# Effects of 2% sodium fluoride solution on the prevention of streptococcal adhesion to titanium and zirconia surfaces

**DOI:** 10.1038/s41598-021-84096-x

**Published:** 2021-02-24

**Authors:** Yukari Oda, Tadashi Miura, Tomoki Hirano, Yoshitaka Furuya, Taichi Ito, Masao Yoshinari, Yasutomo Yajima

**Affiliations:** 1 Department of Oral and Maxillofacial Implantology, Tokyo Dental College, 2-9-18 Kanda misaki-cho, Chiyoda-ku, Tokyo, 101-0061 Japan; 2 Oral Health Science Center, Tokyo Dental College, 2-9-18 Kanda misaki-cho, Chiyoda-ku, Tokyo, Japan

**Keywords:** Peri-implantitis, Implants

## Abstract

Streptococci are associated with dental plaque formation as the early-colonizing bacteria that adhere to titanium (CpTi) and zirconia (TZP) implant abutment surfaces. Effective prevention of peri-implantitis may be possible by removing streptococci as target. This study aimed to evaluate the effects of 2% NaF on the prevention of streptococcal adhesion to CpTi and TZP. After immersion in 2% NaF for 90 min, surface characterization of mirror-polished CpTi and TZP disks were assesed using XPS, EPMA, and SEM. *S. sanguinis*, *S. gordonii*, and *S. oralis* were used as the streptococcal bacterial strains. After 24 h culture, bacterial adhesion was evaluated using an ATP-bioluminescent assay and SEM. In XPS, EPMA, and SEM analyses, fluoride was detected on the CpTi and TZP surfaces after 2% NaF immersion with no signs of localization, and no corrosion on the CpTi disks. Based on the adhesion assay, the adherences of *S. sanguinis*, *S. gordonii*, and *S. oralis* were significantly lower with NaF than without NaF in CpTi (*p* = 0.005, 0.001, and 0.001, respectively) and TZP (*p* = 0.003, 0.002, and 0.001). This was also confirmed by SEM. In conclusion, 2% NaF reduced the adhesion of streptococci to the CpTi and TZP surfaces.

## Introduction

Early-colonizing bacteria (including streptococci) are the primary cause of dental plaque, followed by the late-colonizing pathogenic bacteria^1^. Some late-colonizing bacteria present in the oral biofilms exhibit a higher pathogenic potential than early-colonizing bacteria during plaque formation^2^. Streptococci have a specific temporal and spatial distributions that are crucial for the development of oral biofilms^3^.

Peri-implantitis is defined as a pathological condition that occurs in tissues surrounding dental implants; this condition is characterized by inflammation of the peri-implant connective tissues and progressive crestal bone loss^4^. Dental plaque is thought to be involved in the etiology of peri-implantitis^5^. It is said that peri-implant mucositis is caused by the accumulation of dental plaque on the surface of the abutment or implant^6,7^. Peri-implant mucositis is the precursor of peri-implantitis. It can cause peri-implantitis with extensive peri-implant crestal bone loss if left untreated^8^. The streptococcal species, including *Streptococcus sanguinis*, *Streptococcus gordonii*, and *Streptococcus oralis* have been detected in subgingival and submucosal plaque samples obtained from individuals with peri-implantitis^9,10^. This condition can be potentially prevented if streptococci, which are the target as early-colonizing bacteria, are reduced adhering to dental implant surfaces.

Titanium (CpTi) is a material widely used in oral implant systems. Additionally, TZP (tetragonal zirconia polycrystal) is also a commonly-used material owing to its aesthetics and superior biomechanical properties including a high toughness of fracture and bending strength. In CpTi and TZP, the biofilm-free surface exposed to the oral cavity must be maintained to prevent the development of peri-implantitis^11^.

The bactericidal effects of sodium fluoride (NaF) against *S. mutans* in natural teeth^12^ are well known. NaF solution is used to create CaF_2_ and reduce the acidic attacks from the bacteria. In *S. mutans* and *S. sanguinis*, it was also reported that NaF significantly suppressed biofilm formation on hydroxyapatites disks^13^. In particular, the application of 2% NaF (9000 ppm of F^−^) is a simple and easy method widely used in dental clinics. There are reports on the application of NaF on natural teeth, however, studies of their application on the implant surface are lacking. For example, Barros and de Gouvêa^14^ have reported the effect of fluoridated prophylactic agents against bacteria and on the corrosion resistance of titanium and titanium alloy. Moreover, Fukushima et al.^15^ have revealed the effects of 0.2% neutral NaF on the CpTi surface and against *S. mutans*. Based on a previous study, CpTi corrosion was observed with the application of 0.2% acidic NaF solution, but not with 2% neutral NaF solution^16^. Some studies have shown that 2% NaF solution did not cause CpTi corrosion if the pH was > 6^17,18^. Moreover, the solution exerted bactericidal effects against *S. mutans*^15^. Since the local application of 2% NaF solution has already been used for natural teeth, if it reduces bacterial adhesion, it will be very useful for implant maintenance.

However, to our knowledge, there is no report focused on 2% NaF solution against other streptococci (*S. gordonii*, and *S. oralis*) adhered on CpTi. Likewise, the effects of 2% NaF solution on bacterial adherence to TZP have not been demonstrated so far. The null hypothesis (H0) of this study is that there is no significant difference in streptococcal adhesion between with and without treatment of 2% NaF solution to CpTi and TZP. If NaF can lower streptococcal adhesion, which is considered to be early-colonizing bacteria on the surface of implant materials, it can be a very effective method for preventing peri-implantitis. For verifying this hypothesis, the current study aimed to evaluate whether 2% NaF solution (9000 ppm F^−^) lower the adhesion of *S. sanguinis*, *S. gordonii*, and *S. oralis*. In addition, effects of 2% NaF solution on CpTi and TZP surfaces was also evaluated.

## Results

### Surface Ra and wettability

The Ra values and wettability of the experimental specimens are shown in Table 1. The mean Ra values (± SD) were 0.021 (± 0.003) μm for CpTi and 0.022 (± 0.002) μm for TZP. The contact angles were 68.1° ± 5.3° for CpTi and 68.4° ± 4.8° for TZP, respectively. There were no significant differences in both the Ra values (*p* = 0.72, n = 5) and contact angles (*p* = 0.76, n = 5) between the CpTi and TZP specimens.

**Table 1:** Ra and contact angle.

	Ra (µm)	Contact angle (°)
CpTi	0.021 ± 0.003	68.1 ± 5.3
TZP	0.022 ± 0.002	68.4 ± 4.8

### XPS (X-ray photoelectron spectroscopy) and EPMA (Electron probe microanalysis) analysis

**Figure 1:**
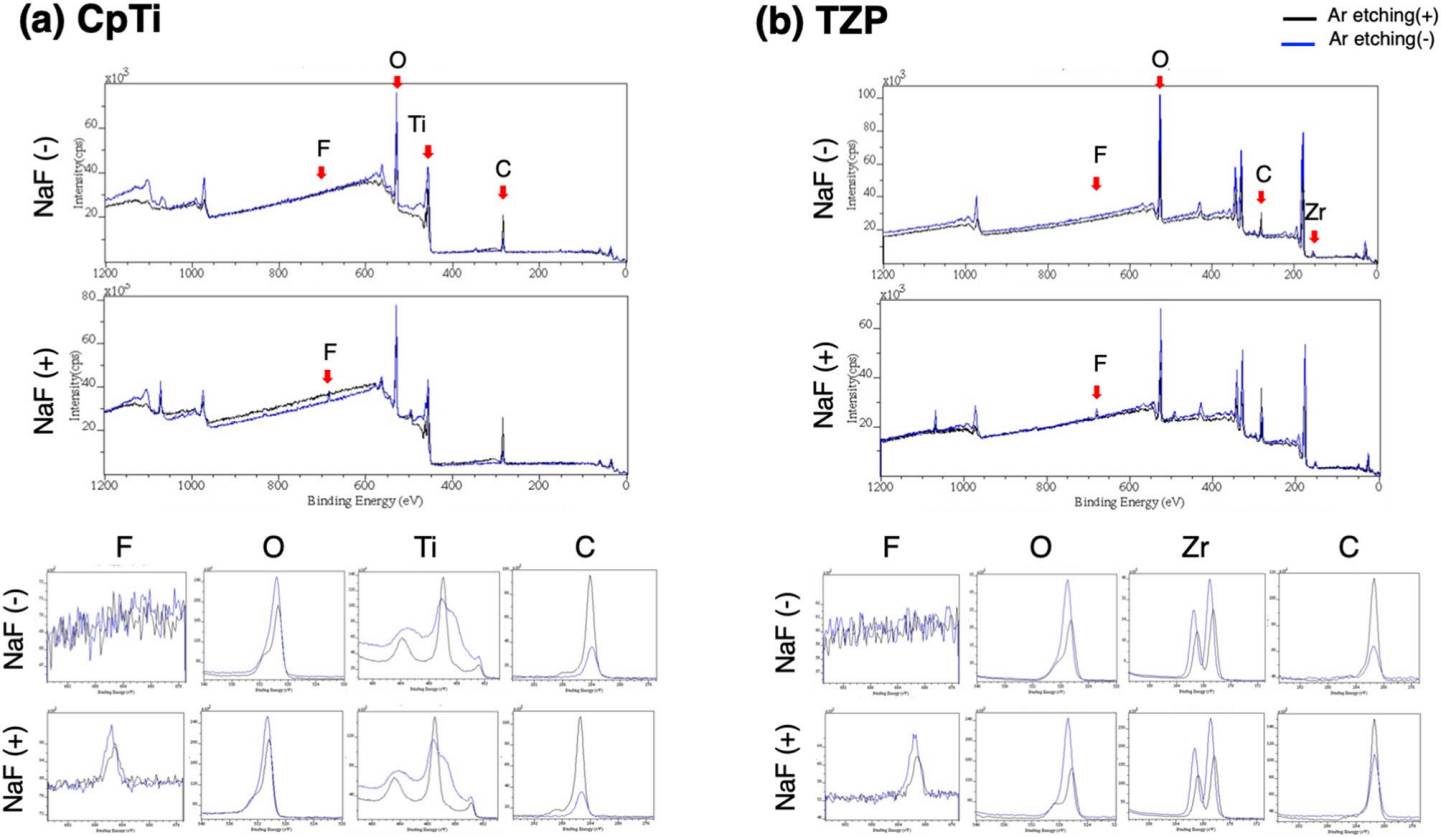
Use of XPS in the analysis of CpTi (**a**) and TZP (**b**). XPS was used to analyze up to a depth of 1 nm from the surface and another 1 nm from the surface with Ar etching. (**a**, **b**) In both CpTi and TZP, F was detected on NaF (+) surfaces, but not in NaF (−) surfaces. With or without Ar etching, F was detected in NaF (+) surfaces, but not in NaF (−) surfaces. The intensity of C decreased, and that of O increased with Ar etching. CasaXPS software version2.3.23 was used (http://www.casaxps.com).

The results of the XPS analyses of the outermost surface of CpTi and TZP are shown in Fig. 1. The samples not immersed in 2% NaF solution are labeled NaF (−), the samples immersed in NaF are labeled NaF (+). In CpTi, with or without Ar etching, F was detected on NaF (+) surfaces, but not in NaF (−) surfaces. Likewise, in TZP, F was detected on NaF (+) surfaces, but not on NaF (−) surfaces. The intensity of C decreased, and that of O increased with Ar etching.

**Figure 2:**
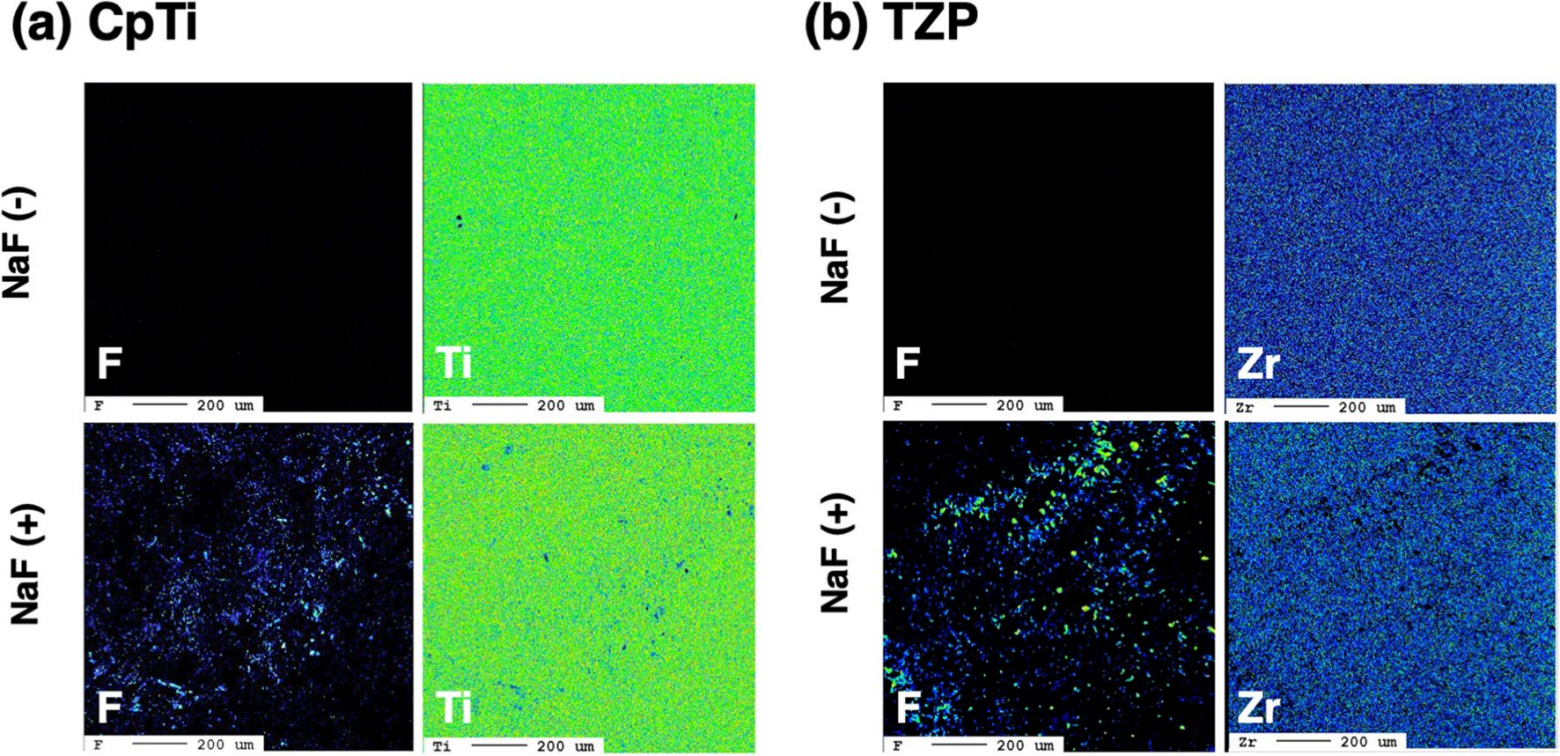
Use of EPMA in the analysis of CpTi (**a**) and TZP (**b**). EPMA was used to analyze up to a depth of 1 μm from the surface. (**a**) F was detected on NaF (+) surfaces, but not on NaF (−) surfaces. However, no signs of localization were observed. Ti was detected on both NaF (+) and NaF (−) surfaces. (**b**) F was detected on NaF (+) surfaces, but not on NaF (−) surfaces. However, no signs of localization were observed. Zr was detected on both NaF (+) and NaF (−) surfaces.

The results of the EPMA analyses of the CpTi and TZP surfaces after immersion in NaF solution are shown in Fig. 2. In CpTi, F was detected on NaF (+) surfaces, but not on NaF (−) surfaces. Moreover, no signs of localization were observed. Ti was detected on both NaF (+) and NaF (−) surfaces. In TZP, F was detected on NaF (+) surfaces, but not on NaF (−) surfaces. However, no signs of localization were observed. Meanwhile, Zr was detected on both NaF (+) and NaF (−) surfaces.

### Analysis of the material surface using SEM

**Figure 3:**
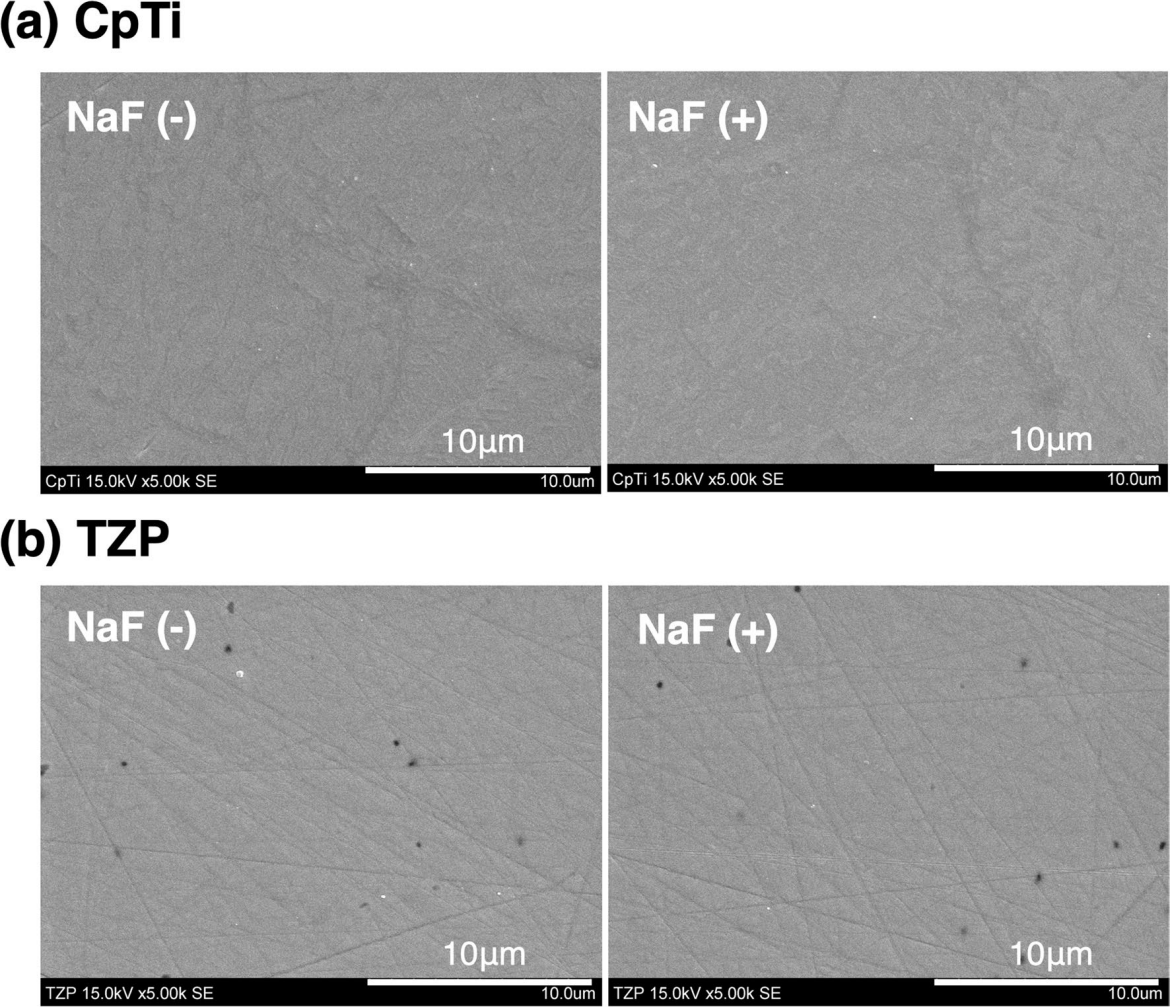
SEM images of the CpTi (**a**) and TZP (**b**) surfaces. In NaF (+) surfaces, SEM images are obtained after immersion in 2% NaF solution. (**a**) In CpTi, there were no changes in NaF (−) and NaF (+) surfaces, and no sign of corrosion was observed. (**b**) In TZP, there were no changes in NaF (−) and NaF (+) surfaces.

In CpTi and TZP, the changes in the material surface caused by immersion in NaF solution are shown in Fig. 3. In CpTi and TZP, there were no changes between the NaF (−) and NaF (+) surfaces. In addition, no signs of corrosion were observed on the surfaces of both materials.

### Adhesion assay

**Figure 4:**
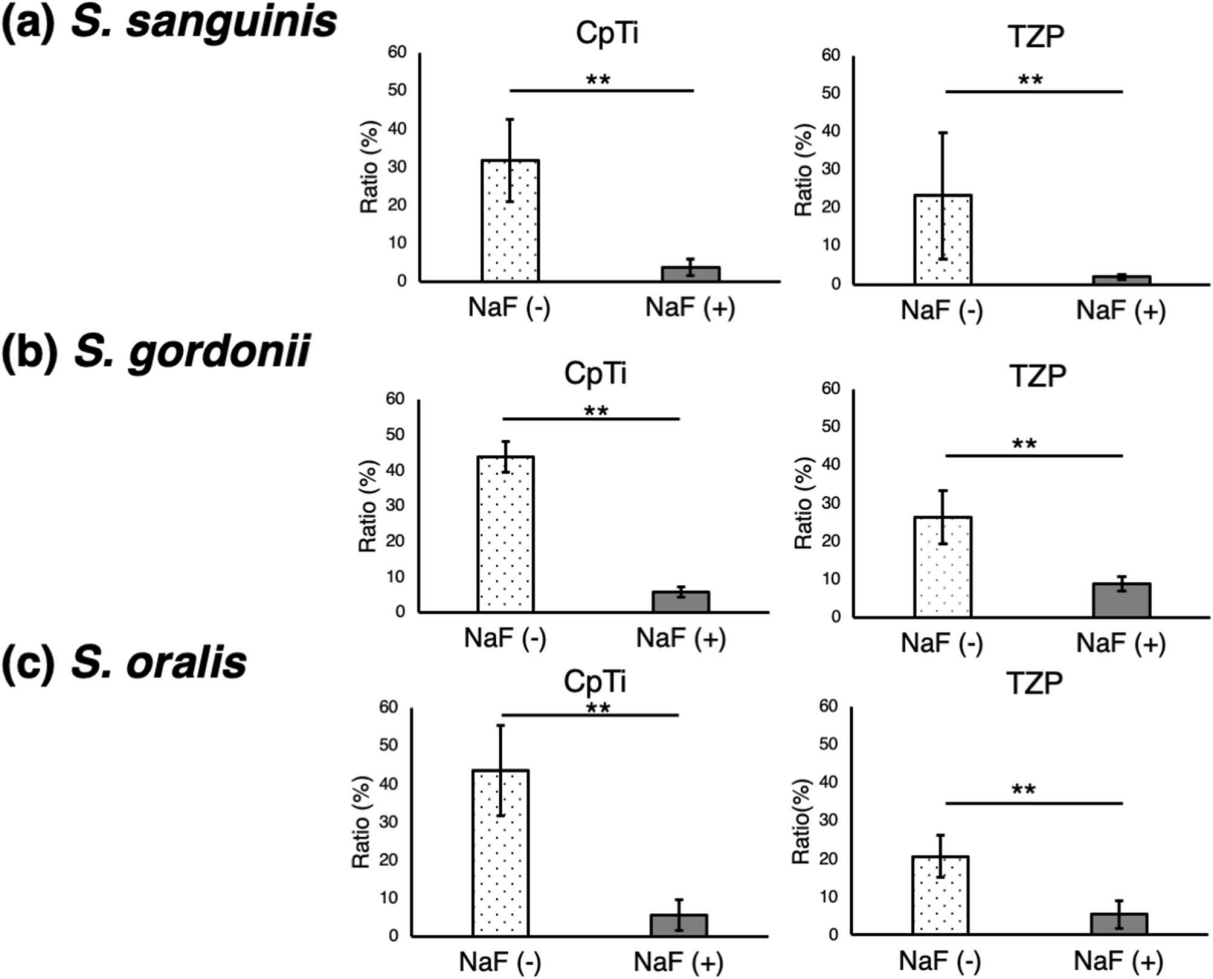
Cell viability of streptococci. The number of streptococci that adhered to the surfaces after 24 h was assessed using the relative light unit (RLU). The vertical axis ratio (%) is defined as the ratio of RLUs on the disks to the initial RLUs of the bacteria liquid cultures. It shows the differences between NaF (+) and NaF (−) surfaces, and they are presented by means (± SD) (**a**, **b**, **c**) On both CpTi and TZP, the cell viability of streptococci on NaF (+) surfaces was significantly lower than that on NaF (−) surfaces. GraphPad Prism version 6.0 was used for statistical analysis (https://www.graphpad.com/scientific-software/prism/).

The cell viabilities of the three streptococcal strains after 24 h were measured based on the relative light unit (RLU), as shown in Fig. 4. The vertical axis ratio (%) is defined as the ratio of the RLU on the disks to the initial RLU of the bacteria liquid culture. Figure 4 shows the differences between NaF (+) and NaF (−) surfaces. They are presented by means (± SD). In CpTi, the cell viabilities of *S. sanguinis*, *S. gordonii*, and *S. oralis* on NaF (+) surfaces was significantly lower than that on NaF (−) surfaces (*p* = 0.005, 0.001, and 0.001, respectively; n = 7 each); similar findings were observed in the TZP specimens (*p* = 0.003, 0.002, and 0.001, respectively; n = 7 each).

### Analysis of bacteria using SEM

**Figure 5:**
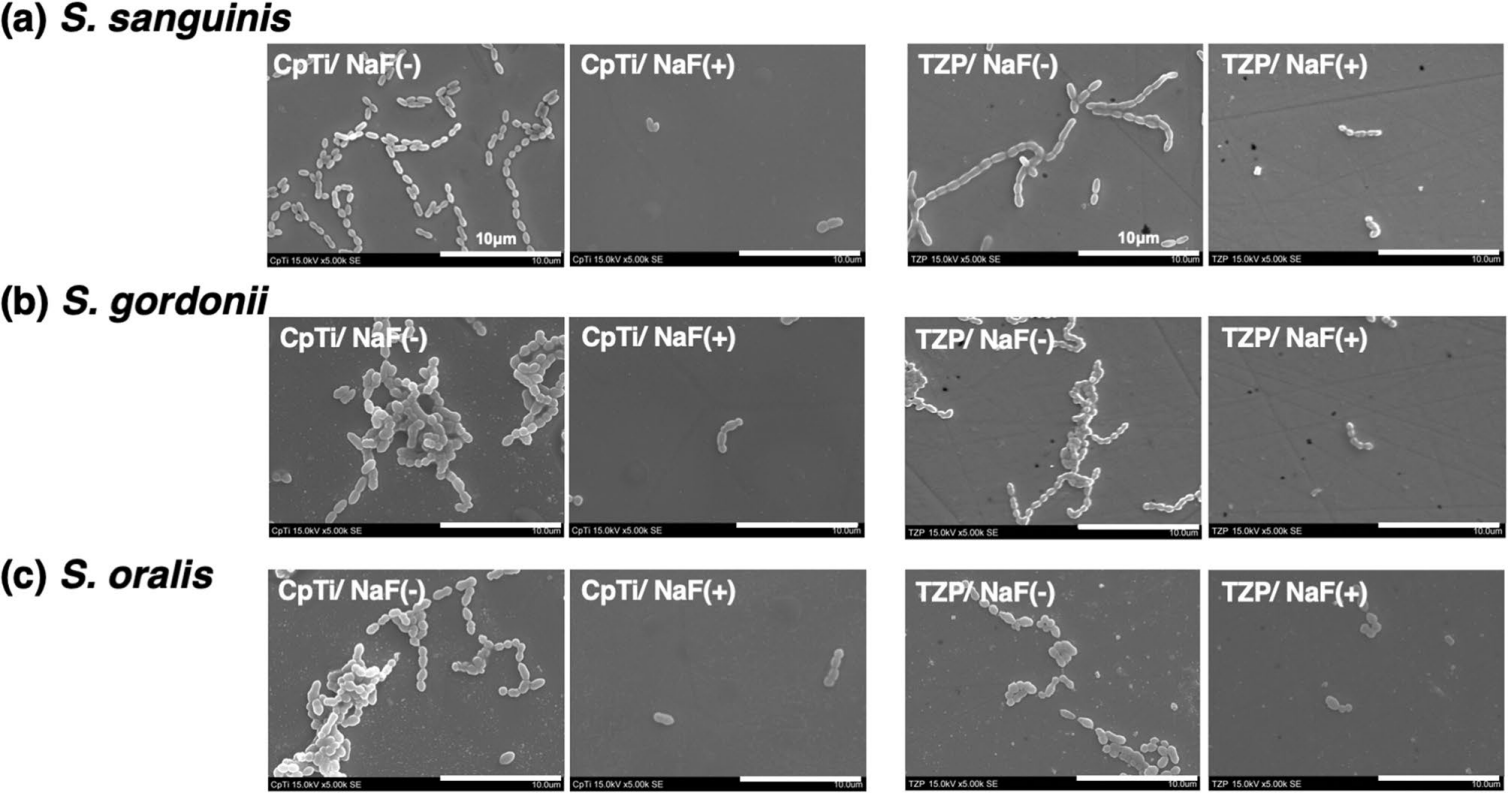
SEM images of the streptococci. The SEM images of the bacteria on the CpTi and TZP surfaces are shown. (**a**, **b**, **c**) In both CpTi and TZP, the streptococcal adherence on NaF (+) surfaces was lower than that on NaF (−) surfaces.

The SEM images of the bacteria on the CpTi and TZP surfaces are shown in Fig. 5. For *S. sanguinis*, *S. gordonii*, and *S. oralis*, the findings of this analysis were similar to those of the cell viability assay. That is, bacterial adherence on NaF (+) surfaces was lower than that on NaF (−) surfaces.

## Discussion

The current study revealed that 2% NaF solution (9000 ppm F^−^) reduced streptococcal adhesion to CpTi and TZP surfaces than without NaF. Therefore, the null hypothesis that there is no significant difference in streptococcal adhesion between with and without treatment of 2% NaF solution to CpTi and TZP was rejected.

Based on the results of the XPS and EPMA analyses, F accumulation was observed on CpTi and TZP with the application of 2% NaF. XPS was used to analyze up to a depth of 1 nm from the surface and another 1 nm from the surface with Ar etching. With or without Ar etching, F was detected on NaF (+) surfaces, but not on NaF (−) surfaces. In addition, EPMA was used to analyze a depth of 1 μm from the surface, and similar results were obtained between analyses using XPS and EPMA. As stated above, accumulation of F up to a depth of 1 nm^–1^ μm on the surface was observed in the NaF treated specimens. In addition, in XPS, the intensity of C decreased, and that of O increased with Ar etching. This result indicates that O and F were present below C. It is generally reported that C and O are detected on the surface of CpTi and TZP when stored in the atmosphere^19–22^. In the current study, C and O were detected on the surfaces of the two specimens, and F was located below C.

No signs of corrosion were observed on the surface of the CpTi in the SEM analysis. The mechanism of CpTi corrosion caused by NaF was as follows^15,16,23,24^:

$$ \begin{aligned} & {\text{NaF}} \to {\text{Na}}^{ + } + {\text{F}}^{ - } \\ & {\text{H}}^{ + } + {\text{F}}^{ - } \to {\text{HF}} \\ & {\text{TiO}}_{2} + 4{\text{HF}} \to {\text{TiF}}_{4} + 2{\text{H}}_{2} {\text{O}} \\ \end{aligned} $$

Hydrogen fluoride (HF) can be formed efficiently from F^−^ when the environmental pH becomes acidic because it can occur with a pKa of 3.2 at 25 °C^23^. Thus, the corrosive effect of HF is based on its concentration and environmental acidity. HF initiates the corrosion of the CpTi surface due to the destruction of the surface oxide layer (TiO_2_)^16,24^, resulting in the production of titanium fluorides, such as TiF_4_^25^. Moreover, the 2% NaF solution does not corrode CpTi surfaces unless the pH was < 6^17,18^. Based on the results of the XPS analysis, the F was not derived from titanium fluorides in the current study; additionally, no corrosion was observed via SEM. The pH value of the gingival crevicular fluid increases with gingival inflammatory symptoms and fluctuates in the range of 6.90 to 8.66^26^. Furthermore, in periodontitis, the subgingival plaque has a neutral pH to alkaline pH and there are many bacterial species that use proteins as nutrient sources, so many metabolites are basic substances that raise the pH, such as ammonia^27,28^. A persistent decrease in pH is thought to be unlikely.

The NaF solution was found to be effective in inhibiting streptococcal adhesion on both CpTi and TZP. Moreover, 2% NaF solution reduced the adhesion of every streptococci specimen on CpTi and TZP surfaces. Several studies have assessed the relationship between fluoride and *S. mutans*, which are cariogenic bacteria. F^−^ is taken up into the cytoplasm through the cell wall by suppressing enolase and glycolysis in the metabolic pathway, thereby reducing intracellular activity^29,30^. As for *S. sanguinis*, *S. gordonii*, and *S. oralis*, although the suppression mechanism is still unknown, F^-^ may be taken up into the cytoplasm through the cell wall. On the other hand, the application of F^−^ is a surface modification technique that can inhibit biofilm accumulation^31^. F^−^-implanted specimens also significantly inhibit the growth of pathogenic bacteria and *S. mutans*; furthermore, they do not influence the corrosion resistance of titanium and the proliferation of mouse-fibroblast cells^32,33^. The primary antibacterial mechanism involves a metal fluoride complex, which acts as an enzyme inhibitor and affects the metabolism of the bacteria^32^. It was reported that metal fluoride complexes are also responsible for the fluoride inhibition of proton-translocating F-ATPases and are thought to act by mimicking phosphate to form complexes with ADP at the reaction centers of the enzymes^32^.

One of the limitations of the current study was that it was performed in vitro. Although 2% NaF solution was used in this study, the actual concentration of F in the saliva might decrease (salivary F clearance)^34^. Thus, suitable conditions for NaF application must be considered in vivo. In one study, 1% NaF suppressed the colonization of *Porphyromonas gingivalis* strains in late-colonizing pathogenic bacteria^35^. Further studies must be conducted to investigate the properties of late-colonizing pathogenic bacteria adherence after early-colonizing bacteria adherence in vitro.

From a clinical point of view, the local application of 2% neutral NaF solution, including F, on the tooth surface could be used to prevent the development of caries in the remaining teeth, even in the presence of CpTi-based abutments. The results of the current study were in accordance with the recommendation that rather than avoiding NaF to protect CpTi-based implant prostheses, it can be applied to protect the remaining teeth. In addition, 2% NaF solution suppresses the adhesion of streptococci species on CpTi- and TZP-based abutments. During maintenance, it is assumed that the oral cavity is cleaned while the abutments at the site of peri-implantitis or peri-implant mucositis are removed and immersed in 2% NaF solution. Efficient implant maintenance might be possible by this method. In addition, streptococci also play a pivotal role as commensals in the oral cavity. Hence, it might be a good strategy to apply NaF locally to the dental abutments rather than to eliminate them from the mouth.

In conclusion, 2% NaF solution lower the adhesion of *S. sanguinis*, *S. gordonii*, and *S. oralis* on the surfaces of both CpTi and TZP specimens. Additionally, F accumulation observed on the surfaces following the application of 2% NaF. No corrosion was observed on the surfaces of the CpTi specimens. Streptococci form a part of the early-colonizing bacteria and initiate the development of peri-implantitis by adhering to the CpTi and TZP abutment surfaces. Thus, a reduction in these bacteria colonies (particularly, streptococci) could lead to a reduction in the attachment of the late-colonizing pathogenic bacteria. The application of a 2% NaF solution might be potentially effective in preventing peri-implantitis.

## Methods

### Specimen preparation

CpTi (grade 2; Kobe Steel, Kobe, Japan) and yttria-stabilized TZP (TZ-3YB-E; Tosoh, Tokyo, Japan) were used. Both CpTi and TZP specimens had a diameter of 13 mm and thickness of 0.5 mm. For polishing of specimens, a polishing machine (Ecomet 3; Buehler, Lake Bluff, IL, USA) was used. First, the CpTi specimens were ground using silicon carbide paper (Aqra; SANKEI, Tokyo, Japan) down to 1,200 grit. Then, they were polished using 3-μm diamond pastes (MetaDi Monocrystalline Suspension 3-μm; Buehler, Lake Bluff, IL, USA) followed by 0.06-μm colloidal silica (MetaDi Monocrystalline Suspension 0.06-μm; Buehler, Lake Bluff, IL, USA). The TZP specimens were ground progressively with 70- and 45-μm diamond disks (DGD 70-μm and DGD 45-μm; Buehler, Lake Bluff, IL, USA). Then, they were finely polished using 9- and 3-μm diamond pastes (MetaDi Monocrystalline Suspension 9-μm and 3-μm; Buehler, Lake Bluff, IL, USA) followed by 0.06-μm colloidal silica. CpTi and TZP specimens were cleaned ultrasonically (Ultrasonic cleaner; Aiwa, Saitama, Japan) and underwent further cleaning with ethyl acetate (Ethyl acetate; Wako, Osaka, Japan), acetone (Acetone; Wako, Osaka, Japan) and distilled water cleaning. Subsequently, the specimens were sterilized in an autoclave (121 °C, 15 min) (HICLAVE HG-80; HIRAYAMA, Saitama, Japan). For at least 24 h until use, they were kept in dry conditions.

### Surface roughness and contact angle

The arithmetic mean surface roughness (Ra), with a length of 4 mm and cutoff value of 0.8 mm, was measured using a three-dimensional measuring laser microscope (LEXT OLS4000; OLYMPUS, Tokyo, Japan). Five specimens from each group were analyzed. The surface wettability of each sample was assessed using double-distilled water using a contact angle meter (Phoenix α; Meiwa-forces, Tokyo, Japan). The contact angles were measured at three different locations on each of the five samples 15 s after the application of the droplets. The drop volume was maintained at 4 μl.

### Immersion in 2% NaF solution

The polished CpTi and TZP specimens were immersed in 2% NaF solution (9000 ppm F^−^, pH = 7.6, 22.5 °C; Fujifilm Wako Pure Chemicals, Osaka, Japan) for 90 min at 37 °C. After immersion, the specimens were removed from the solution. Subsequently, they were stored in dry conditions at 37 °C for at least 24 h until use. The specimens not immersed in 2% NaF solution are labeled NaF (−), the specimens immersed in NaF are labeled NaF (+).

### X-ray photoelectron spectroscopy analysis (XPS)

X-ray photoelectron spectroscopy (XPS) was used to determine the composition and chemical shift of the outermost surface using an X-ray photoelectron spectrometer (AXIS ULTRA; Kratos Analytical, UK), with an X-ray source of Al Kα (monochromator), 15 kV, and 10 mA to identify the intensity of Ti, F, O, and C in the CpTi specimens and Zr, F, O, and C for the TZP specimens with 1 nm depth from the sample surface. The binding energy of each spectrum was calibrated with a C1s of 285.0 eV. Moreover, the elemental analysis of another 1 nm depth from the surface was performed with etching for 10 s using Ar ion at 4.5 kV and 20 mA. Quantitative and curve fitting analyses were performed using a dedicated software (CasaXPS version 2.3.23; Casa Software, Teignmouth, UK; http://www.casaxps.com).

### Electron probe microanalysis (EPMA)

Electron probe microanalysis (EPMA) was performed using an electron probe microanalyzer (EPMA, JXA-8200, JEOL, Tokyo, Japan). Elemental analysis of 1 μm depth from the surface of the specimens was performed at 10 kV and 20 nA electron beam.

### Bacterial strains and culture media

The bacterial species used were *S. sanguinis* ATCC 10,556 (ATCC, American Type Culture Collection, Manassas, VA, USA), *S. gordonii* ATCC 10,558, and *S. oralis* ATCC 35,037. The bacteria were cultured on plates containing brain–heart infusion (BHI; Sigma-Aldrich, St. Louis, MO, USA) and 1.5% agar (Wako, Tokyo, Japan)^36^. Pre-culture was performed in an anaerobic chamber (N_2_: 80%, H_2_: 10%, and CO_2_: 10%) at 37 °C for 1 day　(Anaerobox ANX-5; Hirasawa, Tokyo, Japan). A single colony on the plate was cultured in BHI broth for another 24 h. After liquid culture for another 4 h, a fresh culture was used for the adherence experiment. The optical density of each bacterial suspension was adjusted with BHI broth to 0.2 at 660 nm using a spectrophotometer (Ultrospec 2100 pro; Amersham Biosciences, Piscataway, NJ, USA); it corresponds to 6.0 × 10^7^ CFU/ml. For subsequent experiments, the bacteria were cultured on disks in 24-well plates.

### Cell adhesion assay

Using the adenosine triphosphate-bioluminescent assay, the number of bacteria adhering to the disk surface was measured with a commercial kit (BacTiter-Glo Microbial Cell Viability Assay kit; Promega, Madison, WI, USA). After the incubation of the bacteria for 24 h, all bacterial fluids on the disks were removed and washed with PBS, and an equal volume (200 μl) of BacTiter-Glo reagent was applied to the disk surface. Then, the activity of adenosine triphosphate in the solution was measured using an automatic luminometer (Gene Light Model GL-210A; Microtec, Funabashi, Japan), and the relative luminescence was determined. All assays were performed using three samples of each material. As described previously^37^, n = 7 specimens from each material were used and at least three experiments were performed.

### Analysis of material surface and bacteria using SEM

Based on the analysis using a scanning electron microscope (SEM), the NaF (−) and NaF (+) material surfaces were compared. All specimens were sputter-coated with Au–Pd and were assessed using SEM (JSM-6340F; JEOL, Tokyo, Japan).

The streptococci were cultured as described in the adhesion assay. After the incubation of each type of bacteria, the specimens were fixed with 1.25% glutaraldehyde (Glutaraldehyde Solution; Wako, Osaka, Japan) in phosphate buffered saline (PBS) for 2 h at room temperature. The specimens were then washed three times with PBS and dehydrated using a graded ethanol series (70%, 80%, 90%, 95%, and 100%) (Ethanol; Wako, Osaka, Japan). The specimens were subsequently freeze-dried, sputter-coated with Au–Pd, and seven specimens from each group were assessed using SEM.

### Data analysis

The results are expressed as mean ± standard deviation. Statistical analysis was performed using GraphPad Prism (version 6.0; GraphPad Software, San Diego, CA, USA, https://www.graphpad.com/scientific-software/prism/). Data were analyzed by Shapiro–Wilk test and they were parametric (*p* values < 0.05). They were analyzed using the student’s t-test, and probability *p* values < 0.05 were considered statistically significant.
